# An atypical sarcoidosis involvement in FDG PET/CT: A case report

**DOI:** 10.1097/MD.0000000000005700

**Published:** 2016-12-30

**Authors:** Philippe Robin, Paolo Benigni, Benoit Feger, Pierre-Yves Salaun, Ronan Abgral

**Affiliations:** a Département de Médecine Nucléaire, EA3878 (GETBO) IFR 148, Centre Hospitalier Régional Universitaire de Brest, France; b Département d’Anatomie Pathologique, Centre Hospitalier Régional Universitaire de Brest; c Service d’Oto-Rhino-Laryngologie, Clinique Pasteur, Brest, France.

**Keywords:** extrapulmonary localization, fluorodesoxyglucose, larynx, positron emission tomography, sarcoidosis

## Abstract

**Rationale:**

Sarcoidosis is an idiopathic systemic inflammatory granulomatous disorder comprised of epithelioid and multinucleated giant cells with little necrosis which involve various organs. Laryngeal involvement is extremely rare, with a prevalence of about 0.5 to 1%.

**Diagnoses:**

Here we present a case of laryngeal involvement of sarcoidosis demonstrated on ^18^F-Fluorodesoxyglucose Positron-Emission Tomography/Computed Tomography (FDG PET/CT).

**Patient concerns:**

A 63 year-old man suffering from dysphonia was referred to our department for characterization of laryngeal lesion suspicious for cancer with non-informative biopsy, the sample was not sufficient for diagnosis.

**Interventions:**

FDG PET/CT showed a pathological uptake on the right vocal cord, but also highlighted a bilateral uptake in intrathoracic hilar lymphadenopathy areas, typically found in several inflammatory diseases.

**Outcomes:**

New laryngeal targeted biopsies revealed non-caseating epithelioid granulomas suggesting sarcoidosis involvement. After 6 months of systemic steroid treatment, FDG PET/CT showed a significant decrease of the laryngeal uptake.

**Lessons:**

This case shows the usefulness of FDG PET/CT to accurately assess inflammatory activity in rare extra-pulmonary sarcoidosis involvement. Moreover, this case emphasizes that FDG PET/CT is an interesting tool for assessing therapeutic efficacy of inflammatory diseases such as sarcoidosis.

## 1 Introduction

Sarcoidosis is an idiopathic systemic inflammatory granulomatous disorder comprised of epithelioid and multinucleated giant cells with little necrosis.^[1]^ The exact cause of sarcoidosis is still unknown. It commonly affects young and middle-aged adults and frequently presents with bilateral hilar lymphadenopathy and pulmonary infiltration. However, sarcoidosis can involve any organ in the body. Laryngeal involvement is extremely rare, with a prevalence of about 0.5% to 1%.^[2]^ Here, we present a case of laryngeal involvement of sarcoidosis demonstrated on ^18^F-fluorodeoxyglucose positron-emission tomography/computed tomography (FDG PET/CT).

## 2 Case presentation

In October 2014, a 63-year-old man suffering from dysphonia was referred to our nuclear medicine department for characterization of laryngeal lesion suspicious for cancer shown by laryngoscopy, with noninformative biopsy, the sample was not sufficient for diagnosis.

**Figure 1:**
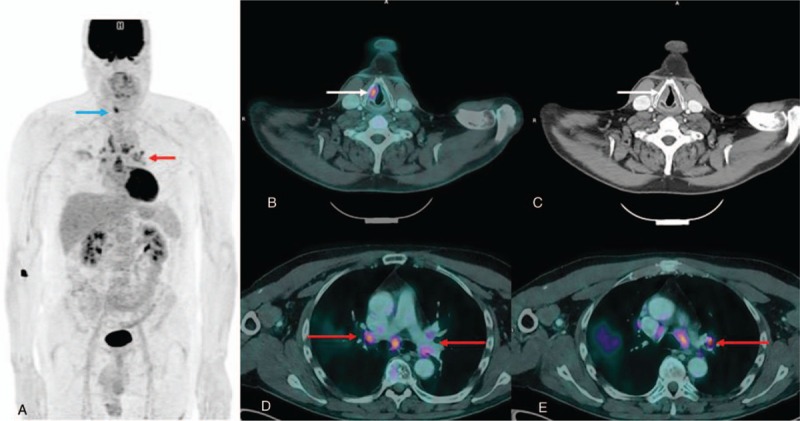
FDG PET/CT maximum intensity projection and FDG PET/CT axial slices images at diagnosis. Images showed a pathological FDG uptake in the right upper aerodigestive tract (a, blue arrow), with a right vocal cord thickening (b, c, white arrows), without cervical nodal abnormality. Moreover, images demonstrated a bilateral and symmetric abnormal uptake in intrathoracic hilar lymphadenopathy (SUV_max_ = 6.1) (d, e, red arrows). FDG PET/CT = ^18^F-fluorodesoxyglucose positron-emission tomography/computed tomography, SUV_max_ = maximum standardized uptake value.

FDG PET/CT maximum intensity projection showed a pathological uptake in the right upper aerodigestive tract evocative of cancer, without cervical nodal abnormality. FDG PET/CT axial slices showed an abnormal uptake in a right vocal cord thickening, with an unusual high metabolic activity (maximum standardized uptake value, SUV_max_ = 9) suggested the possibility of a primary laryngeal tumor, less likely an infectious or inflammatory disease. Moreover, FDG PET/CT axial slices also showed a bilateral and symmetric abnormal uptake in intrathoracic hilar lymphadenopathy (SUV_max_ = 6.1) typically found in inflammatory diseases such as sarcoidosis (Fig. 1).

**Figure 2:**
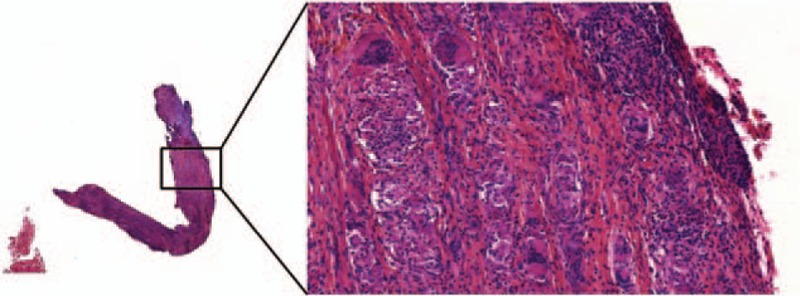
Histopathology of laryngeal biopsy sample. Histopathology revealed noncaseating epithelioid granulomas, characteristic finding of sarcoidosis involvement.

Multiple laryngeal biopsy samples revealed noncaseating epithelioid granulomas, characteristic finding of sarcoidosis involvement (Fig. 2). Differential diagnoses such as infectious diseases (negative culture for mycobacterium and fungi) or tumor-related sarcoïd reaction were excluded.

**Figure 3:**
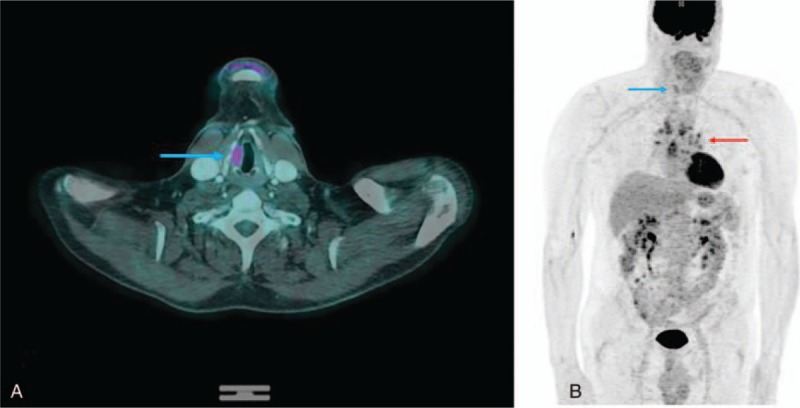
FDG PET/CT images after 6 months of treatment. FDG PET/CT images showed a significant decrease (SUV_max_ = 4.5) of the abnormal uptake in the right vocal cord (a,b, blue arrow). Stable uptake in intrathoracic hilar lymphadenopathy (SUV_max_ = 6.5) (b, red arrow). FDG PET/CT = ^18^F-fluorodesoxyglucose positron-emission tomography/computed tomography, SUV_max_ = maximum standardized uptake value.

Patient was treated with systemic steroid treatment. After 6 months of treatment, the patient underwent FDG PET/CT for therapeutic response assessment in April 2015. FDG PET/CT showed a significant decrease of the abnormal uptake in the right vocal cord (SUV_max_ = 4.5) and confirmed the benign etiology of the lesion; moreover, FDG PET/CT showed a stable uptake in intrathoracic hilar lymphadenopathy (SUV_max_ = 6.5) (Fig. 3). Patient informed consent was given.

## 3 Discussion

Sarcoidosis is an idiopathic systemic inflammatory granulomatous disorder comprised of epithelioid and multinucleated giant cells with little necrosis.^[1]^ It commonly affects young and middle-aged adults and frequently presents with bilateral hilar lymphadenopathy and pulmonary infiltration. The eyes, skin, liver, spleen, lymph nodes, salivary glands, heart, nervous system, muscles, bones, and other organs may also be involved. Laryngeal involvement is extremely rare, with a prevalence of about 0.5% to 1%.^[2]^ The three criteria for its diagnosis are: clinical and radiological presentation, evidence of noncaseating granulomas, and evidence of no alternative diseases. Granulomas of known causes such as infectious diseases (mycobacterial, fungal, or parasitic infections), chronic inflammatory diseases such as pneumoconiosis, Wegener's granulomatosis, or tumor-related sarcoïd reaction, must be excluded. Extrapulmonary localizations should be detected with a thorough physical examination, with a systematic ophthalmological examination, and with an electrocardiogram and blood tests. Confidence in diagnosis can be strengthened with time.^[3]^

FDG PET/CT is routinely used for the diagnosis, staging, and therapeutic assessment of several malignancies.^[4]^ Due to the correlation between accumulation of FDG and glucose transporter type 1 cell membrane of activated granulocytes overexpression,^[5]^ it becomes nowadays a relevant tool for the management of several infectious and inflammatory diseases, such as sarcoidosis.^[6–8]^ Previous studies have shown that FDG PET/CT can be used to accurately assess inflammatory activity in patients with persistent symptoms without biological inflammatory activity, especially in uncommon localizations or when a biopsy is not possible.^[9]^ FDG PET/CT could be the most reliable method for the detection of granulomatous myocardial lesions with appropriate diet without carbohydrates which inhibit physiological myocardial uptake.^[10]^

## 4 Conclusion

This case confirms the usefulness of FDG PET/CT in sarcoidosis disease especially for the diagnosis of potentially rare extra-pulmonary involvement. Moreover, this case emphasizes that FDG PET/CT is an interesting tool for assessing therapeutic efficacy of inflammatory diseases.
